# Solid-state NMR Study of Ion Adsorption and Charge Storage in Graphene Film Supercapacitor Electrodes

**DOI:** 10.1038/srep39689

**Published:** 2016-12-21

**Authors:** Kecheng Li, Zheng Bo, Jianhua Yan, Kefa Cen

**Affiliations:** 1 State Key Laboratory of Clean Energy Utilization, Institute for Thermal Power Engineering, College of Energy Engineering, Zhejiang University, Hangzhou, Zhejiang Province, 310027, China

## Abstract

Graphene film has been demonstrated as promising active materials for electric double layer capacitors (EDLCs), mainly due to its excellent mechanical flexibility and freestanding morphology. In this work, the distribution and variation pattern of electrolyte ions in graphene-film based EDLC electrodes are investigated with a ^11^B magic-angle spinning nuclear magnetic resonance (MAS-NMR) spectroscopy. For neutral graphene films soaked with different amounts of electrolytes (1 M TEABF_4_/ACN), weakly and strongly adsorbed anions are identified based on the resonances at different ^11^B chemical shifts. Unlike other porous carbonaceous materials, the strongly adsorbed anions are found as the major electrolyte anions components in graphene films. Further measurements on the ion population upon charging are carried out with applying different charging voltages on the graphene films. Results indicate that the charging process of graphene-film based EDLCs can be divided into two distinct charge storage stages (*i.e.*, ejection of co-ions and adsorption of counter-ions) for different voltages. The as-obtained results will be useful for the design and fabrication of high performance graphene-film based EDLCs.

Electrochemical double layer capacitors (EDLCs), also known as supercapacitors or ultracapacitors, have attracted considerable attention due to their high power density, long cycle life and low maintenance cost^1^,^2^,^3^. While extensive efforts have been devoted to the design and construction of high performance active materials, it is equally important to explore the underlying charge storage mechanism of EDLCs^4^,^5^,^6^,^7^. A number of theoretical and experimental methods have been developed to investigate the structure and dynamics of electrode-electrolyte interface in EDLCs^8^,^9^,^10^,^11^,^12^. Typically, molecular dynamic (MD) simulation is capable of describing the movement and adsorption of ions, as well as the formation of micro-structures of EDLCs at the active materials and current collector interfaces, based on the calculation at molecular/atomic scales^13^,^14^,^15^; *in situ* electrochemical quartz crystal microbalance (EQCM) is highly effective at determining the mass variation of electrode within charging-discharging processes, by measuring the change in frequency of a quartz crystal resonator^11^,^16^; and *in situ* infrared spectroscopy has been employed to experimentally monitor the migration of electrolyte ions within the pores of porous electrodes^12^.

In recent years, nuclear magnetic resonance (NMR) has emerged as an element-selective, highly localized and quantitative technique to obtain the atomic-scale information on the local environments with direct observation^8^,^17^. As for EDLCs employing carbonaceous active materials, the species close to the solid surfaces experience a diamagnetic shielding (arising from the “ring current” in carbon electrodes^18^,^19^,^20^), leading to the shift of NMR signals to low frequencies. This chemical shift, *i.e.*, the so-called nucleus-independent chemical shift (NICS), can be used as a fingerprint to distinguish the adsorbed species (ions and molecules) from the free ones in the bulk electrolyte^17^,^18^.

NMR studies have been applied to porous materials for the understanding of the adsorption of molecules in micro- and nano-sized pores. Harris *et al*. reported the adsorption of water and phosphorus-containing compounds on activated carbons^21^,^22^,^23^,^24^, where distinct resonances corresponding to the adsorbed and non-adsorbed molecules were observed. Similar observation was also presented for the adsorption of water^25^,^26^, hydrogen^26^,^27^, and methanol^28^ inside carbon nanotubes (CNTs). Forse *et al*.^29^ and Borchardt *et al*.^30^ unveiled the effects of relative pore/ion size on the adsorption of tetraethylammonium (TEA^+^) and tetrafluoroborate (BF_4_^−^) in carbide-derived carbons and other porous carbon materials of well-defined, variable pore sizes. With *ex situ* magic-angle spinning (MAS) NMR, comprehensive information on the anions, cations, and remaining solvent molecules inside or outside the porosity of active carbons were provided by Deschamps *et al*.^31^ Moreover, based on the real-time observation on the charging process of EDLCs employing TEABF_4_ in acetonitrile (ACN) electrolyte, Wang *et al*.^20^,^32^ presented the migration of ions between the electrodes and the changes in the nature of ion binding to the surface. Such an *in situ* measurements were also conducted on carbons soaked in different organic^33^,^34^ and aqueous electrolytes^35^,^36^. The above NMR studies provided useful insights into the molecular mechanisms of the charge storage of EDLCs, including the adsorption of counter-ions into the electrode pores, expulsion of co-ions form pores, and ion exchange between anions and cations^8^,^17^,^37^,^38^,^39^.

Graphene is a two-dimensional nanomaterial with huge specific surface area and a series of unique physical, chemical and mechanical properties^40^,^41^. Thin film made of graphene stacks, also known as “graphene paper” or “graphene film”, has attracted great attention for EDLCs, mainly due to its excellent mechanical flexibility and freestanding morphology^42^,^43^. MD simulation^10^,^44^,^45^,^46^ and DFT calculation^44^,^45^ were conducted to explore the charge storage mechanisms. Typically, the influences of temperature^46^, electrolyte chemical structure^45^, and applied voltage^46^ on the electric double layer structures and capacitance of EDLCs were unveiled with MD simulation. In our recent works, the crucial roles of channel width^10^, edge effect^44^, and charge density^10^ on EDLCs performance were investigated with experimental research and numerical simulations. Despite the motion of particles has been well described at molecular/atomic scale with the above theoretical simulations, a direct observation on the ion distribution and ion population upon charging in graphene films is highly needed.

In this work, solid-state ^11^B MAS-NMR spectroscopy was applied to graphene-film based EDLCs using TEABF_4_/ACN electrolytes. The adsorption behaviors of BF_4_^−^ anions in neutral graphene films with different electrolyte feedings were investigated. Graphene-film based EDLCs were then assembled and the changes in the population of anions with different adsorption states in electrode materials were measured at different charging voltages. The as-obtained results provide useful insights into the charge storage mechanisms in graphene nano-channels, which could be helpful in designing high-performance EDLCs.

## Results

**Figure 1:**
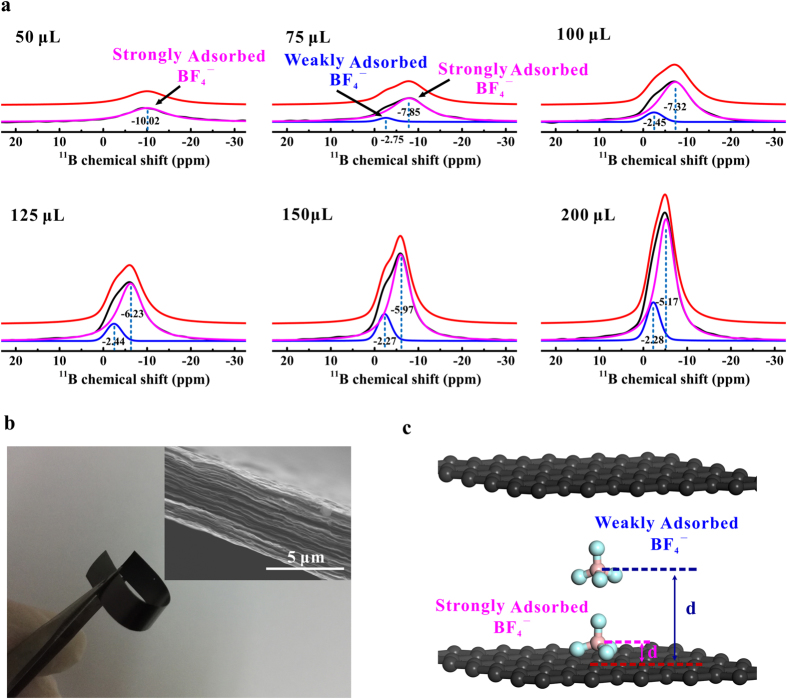
(**a**) ^11^B MAS-NMR spectra of graphene films (60 mg) soaked with different amounts (50~200 μL) of 1 M TEABF_4_/ACN electrolyte. (**b**) Digital photograph of graphene films. Inset: Cross-section scanning electron microscope (SEM) image of the graphene materials. (**c**) Schematic of the weakly and strongly adsorbed sites in graphene nano-channel.

Figure 1a shows the ^11^B MAS-NMR spectra of graphene films soaked with different amounts (50~200 μL) of 1 M TEABF_4_/ACN electrolytes. Each spectrum is fitted with mixed Gaussian/Lorentzian lineshapes, where the as-obtained NMR spectra are shown in black, the fitted components are shown in dark blue and purple, and the total fitted lineshapes are shown in red. At a relatively low electrolyte loading of 50 μL, a single resonance was detected at the ^11^B chemical shift of −10.02 ppm. With the increase of the electrolyte loading, this resonance increased in intensity and shifted in peak position to higher frequencies (*i.e*., the left-hand side of axis), reaching to −5.17 ppm with the electrolyte loading volume of 200 μL. Meanwhile, a second resonance at higher frequency emerged with the electrolyte loading volumes lager than 75 μL. The chemical shift of the second resonance (−2.75~−2.28 ppm) is close to that of crystalline TEABF_4_ (−1.13 ppm, see Fig. S1 in Supplementary Information).

The different chemical shifts of the resonances provide an indication of their origin in graphene materials^29^. As for the current graphene films with layered structure (see Fig. 1b), different adsorption sites can be specifically mapped to the positions on the graphene nano-channel surfaces with different adsorption states (see Fig. 1c). As is well known, the resonances corresponding to adsorbed species on carbon surface shift to low frequency as compared to free species, due to the diamagnetic contribution of the “ring current” in the carbon^17^. Consequently, the deconvoluted peaks at low frequencies (*e.g*., −10.02 ppm for electrolyte loading volume of 50 μL) arise from the ^11^B atoms of strongly bound BF_4_^−^ anions on carbon surface, which is herein referred to as “strongly adsorbed state”. Density functional theory (DFT) calculation indicates that the “ring current” effects on adsorbed ions increased rapidly with the decreasing ion-surface distance, which is strongly related to the size of graphene domains, pore width, and curvature of carbon surface^18^,^32^. On the other hand, the resonances at higher frequencies (*e.g*., −2.75 ppm for electrolyte loading volume of 75 μL) are assigned to the weakly bound BF_4_^−^ anions. These anions situated further from the graphene surfaces, corresponding to the “weakly adsorbed state”. The anions at this position were weakly affected by the graphene surfaces and therefore presented a chemical shift close to that of crystalline TEABF_4_. Due to the rapid dynamic exchange of electrolyte ions between the weakly and strongly adsorbed sites on the NMR timescale^30^,^31^, the observed spectrum is an averaging chemical shift for the anions at different states.

**Figure 2:**
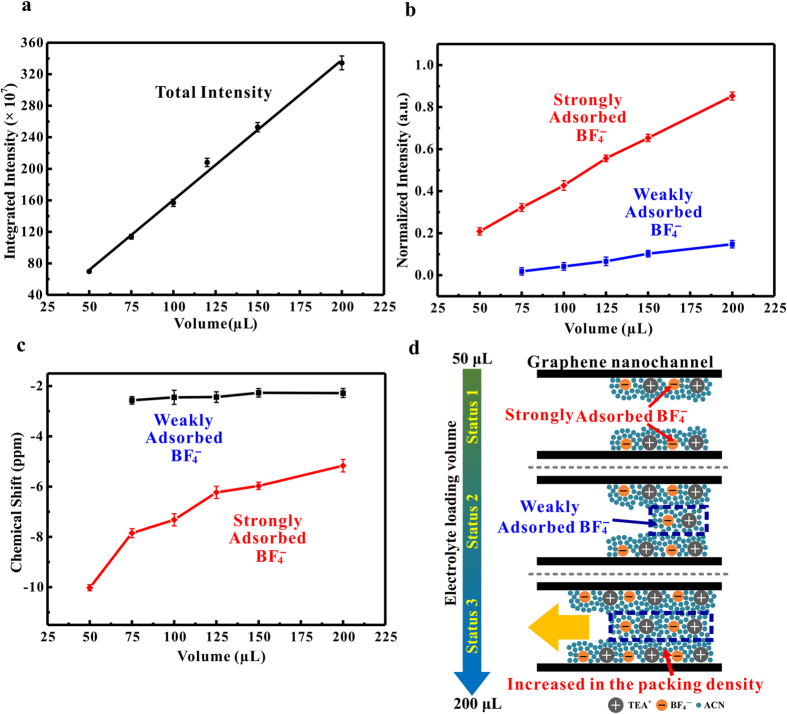
(**a**) The integrated intensity of experimental NMR spectra as a function of electrolyte loadings. (**b**) Deconvoluted intensity and (**c**) chemical shifts of weakly and strongly adsorbed resonances at different loading volumes. (**d**) Schematic illustrations of possible wetting process of graphene films.

Figure 2a shows the integrated intensity of experimental NMR spectra as a function of electrolyte loadings. A quasi-lineal relation between the total intensity and BF_4_^−^ anion population in graphene film was obtained, providing a relationship to quantify the absolute number of ions for a given resonance intensity in the NMR spectrum. Figure 2b shows the deconvoluted intensities of the weakly and strongly adsorbed resonances for different electrolyte loadings. Each value was normalized by the total intensity of the graphene films soaked with 200 μL 1 M TEABF_4_/ACN. Both the strongly and weakly adsorbed resonances intensities increased with the increasing electrolyte loadings. For each certain electrolyte loading, the strongly adsorbed resonances presented a higher intensity than that of the weakly adsorbed resonances. This observation is different from previous NMR results on some other porous carbon materials, where the weakly adsorbed (*i.e*., ex-pore) resonances presented a higher intensity than the strongly adsorbed (*i.e*., in-pore) counterparts^29^,^31^,^32^. It can be attributed to the unique ion adsorption behaviors in graphene nano-channels. As for the activated carbon electrode materials (*e.g*., prepared by mixing 95 wt% carbon powder with 5 wt% PTFE^20^,^32^,^33^,^34^), the “in-pore” resonances arise from the adsorbed ions on carbon surface within micropores, and the “ex-pore” resonances are assigned to the ions in reservoirs of electrolyte in large space between primary carbon particles. As a consequence, the number of “in-pore” ions is less than that of the “ex-pore” ions, leading to a relatively higher intensity of the weakly adsorbed resonances. In contrast, the graphene films used in the current work are binder-free materials with a unique two-dimensional layered structure. Both the weakly and strongly adsorbed anions are mainly located inside the well-defined nano-channels between adjacent graphene sheets. It is reasonable that the strongly adsorbed anions are the major component of anions in graphene films, which could play a leading role in charge storage process of graphene-film based EDLCs.

Figure 2c shows the changes of chemical shift for the weakly and strongly adsorbed resonances with the increasing electrolyte loadings. The chemical shifts of the weakly adsorbed resonances were almost kept as a constant. The increasing chemical shifts of the strongly adsorbed resonances could be attributed to the exchange of strongly bound anions on the graphene surface with the weakly bound anions outside the adsorption layer^20^. Since the NMR timescale is much longer than the timescale of ion exchange, the fast exchange of electrolyte anions between the weakly and strongly adsorbed sites makes these subpopulations resolved in the NMR spectrum^47^. With the increasing electrolyte loading volumes, the number of anions involved in exchange increased gradually (as shown in Fig. 2b), leading to a time-averaged ion-distribution in the pore volume of graphene films^23^,^48^. Consequently, the measured chemical shifts of BF_4_^−^ anions were the NICS averaged over the pore space and had a less negative value than that of anions adsorbed on graphene surfaces^48^.

Figure 2d schematically shows the ion adsorption process within the graphene channels, based on the analysis on the NMR spectra. For the relatively low electrolyte loadings of less than 75 μL, the absence of weakly adsorbed resonances indicates that the BF_4_^−^ anions are preferentially adsorbed on the graphene surface (as schematically shown in the “status 1”). This phenomenon could be attributed to the high adsorption ability of graphene^48^. Similar observation was also reported in the NMR study on the adsorption of methanol on CNTs^28^. The weakly adsorbed anions were discovered at the electrolyte loading volume of 75 μL (as schematically shown in the “status 2”). However, it is different from the observation in previous NMR studies that the weakly (*i.e*., the ex-pore) adsorption does not occur until the strongly (*i.e*., the in-pore) adsorption has attained saturation^17^. In the current work, the strongly adsorbed resonances intensity did not reach a maximum value (*i.e*., the strongly adsorption was not saturated), when the weakly adsorbed anions were observed. Both the weakly and strongly resonances intensities increased with the increasing electrolyte loadings, indicating that the strongly and weakly adsorption were carried out simultaneously. For the relatively high electrolyte loadings of more than 75 μL, the intensities of both the strongly and weakly adsorbed resonances increased as a function of electrolyte loadings, indicating that the number of BF_4_^−^ anions in this two adsorption sites increased gradually. This observation could be attributed to two possible adsorption processes of electrolyte ions. With the increase of electrolyte loadings, the electrolyte ions entered into the inner space of the graphene channels, and the volume occupied by the electrolyte ions increased gradually. Meanwhile, the packing density of the ions within graphene nano-channels increased with the increasing electrolyte loadings, and therefore more ions were stockpiled in graphene films (as schematically shown in the “status 3”).

To further describe the ion packing behavior during the adsorption process, a coefficients *η* was introduced:


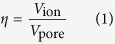


where *V*_ion_ and *V*_pore_ represent the volume of electrolyte ions and the pore volume of graphene film, respectively. According to N_2_ adsorption-desorption isotherms, the graphene film used in the current work has a pore volume (*V*_pore_) of 2.11 cm^3^ g^−1^, which is in agreement with the results of other researches^49^,^50^.

Assuming that there are equal numbers of anions and cations in graphene film, the total volume occupied by full-solvated electrolyte ions, *V*_ion_, can be calculated as:


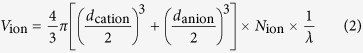


where *d*_cation_ represents the diameter of the full-solvated cations (TEA^+^), *d*_anion_ represents the diameter of the full-solvated anions (BF_4_^−^), and *λ* refers to the packing factor of full-solvated electrolyte ions. For TEABF_4_/ACN electrolyte, *d*_cation_ and *d*_anion_ are 1.3 nm (TEA^+^) and 1.16 nm (BF_4_^−^), respectively^51^. Since the value of *d*_cation_ nearly equals to that of *d*_anion_, the minimum value of *V*_ion_ can be estimated by considering a maximum packing density of solvated anions and cations (for equal spheres, the packing factor *λ* has a range from 52.36 to 74.05%)^52^. The numbers of anions and cations per gram of graphene film at each loading volume can be calculated by the following equation:


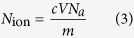


where *c* is the electrolyte concentration, *V* represents the loading volume, *N*_*a*_ refers to the Avogadro’s number and *m* is the sample weight.

For the electrolyte loading volumes of 50 and 75 μL, the coefficients *η* were calculated as 0.63 and 0.95, respectively. These values are less than 1, which means that the volumes occupied by full-solvated electrolyte ions are less than the pore volume of graphene film. The coefficients *η* were calculated as 1.3, 1.6, 1.9 and 2.5 at the electrolyte loading volumes of 100, 125, 150 and 200 μL, respectively. It suggests that the total volume occupied by electrolyte ions is higher than the pore volume of graphene films, indicating the partial desolvation of ion solvation shells during the adsorption process.

**Figure 3:**
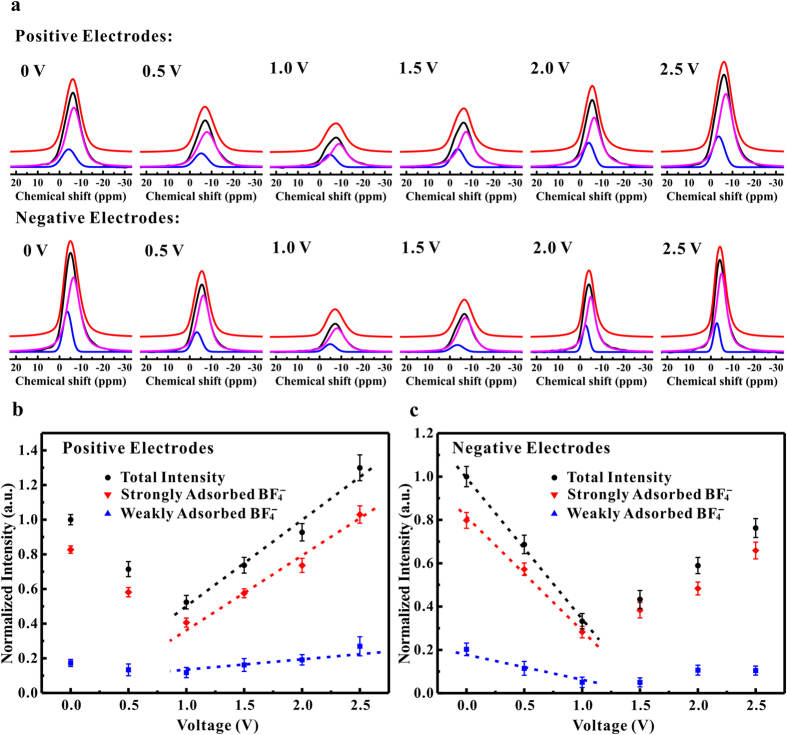
(**a**) ^11^B MAS-NMR spectra of positive and negative electrodes at different charging voltages. (**b**~**c**) The Intensities of the strongly and weakly adsorbed resonances in the positive and negative electrodes at different charging voltages.

Figure 3a shows the ^11^B MAS-NMR spectra of positive and negative electrodes at different charging voltages. For the positive electrodes, the peak intensity of NMR lines first decreased and then increased with an increasing applied voltage from 0 to 2.5 V. Two resonances corresponding to weakly and strongly adsorbed BF_4_^−^ anions were unveiled by deconvolution of these as-obtained NMR spectra. The strongly adsorbed resonances played a predominant role on the charging process. For the negative electrodes, similar observations were also obtained upon charging.

Figure 3b presents the fitted intensity of strongly and weakly resonances for positive electrodes, based on the deconvoluted NMR spectra. The total intensity of experimental NMR spectrum at 0 V was used as a reference. An abnormal decrease in the intensity of strongly adsorbed resonances was observed between 0 and 1.0 V, which means a number of BF_4_^−^ anions were departed from the graphene channel during the charging process. It suggests that the charge storage in graphene electrode materials was not driven by the adsorption of anions as the traditional theory supposed^17^. In this case, the ejection of TEA^+^ cations was the primary mechanism for the charging process. At the charging voltage higher than 1.0 V, an abrupt increase in the intensity of strongly adsorbed resonances was observed. It indicates that a large number of BF_4_^−^ anions were adsorbed into the graphene nano-channels upon charging. Therefore, the adsorption of anions played a more significant role in charge storage between 1.0 and 2.5 V. Similar trends were observed for the negative electrodes (see Fig. 3c). When the charging voltages was lower than 1.0 V, a rapid decrease in intensity of the strongly adsorbed resonances showed up. It suggests that a large number of strongly adsorbed BF_4_^−^ anions were expulsed from the graphene nano-channels and that this played a more significant role in the charge storage process. As the charging voltage increase from 1.0 to 2.5 V, the abnormal increase in intensity of the strongly adsorbed resonances indicates that a number of BF_4_^−^ anions enter into the graphene channels during charging. However, for the negative electrodes, surplus cations were supposed to counteract the negative charge that was developed on the electrode surface. As a consequence, the mechanisms of charge storage was dominated by the adsorption of TEA^+^ cations in the voltage range operated in the current work.

## Discussion

The results presented in this study highlight the complexity of charge storage mechanisms within the graphene nano-channels. To better understand the charge storage process that had taken place in graphene films upon charging, the electronic charge and ionic charge stored on the positive electrodes were calculated respectively.

**Figure 4:**
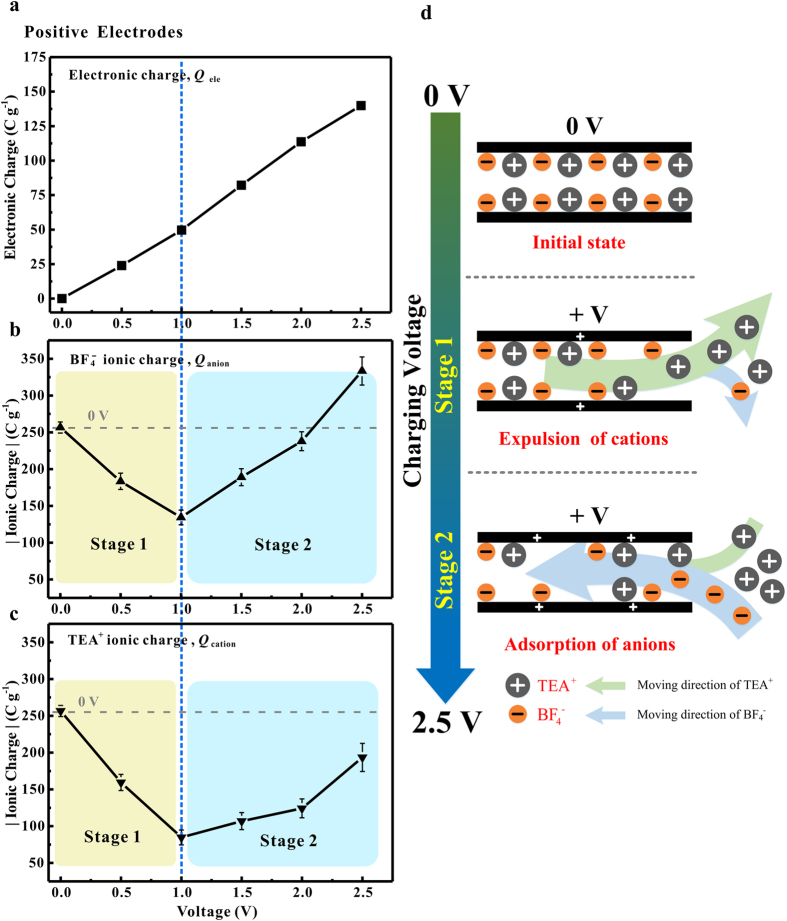
(**a**) The electronic charge, (**b**) BF_4_^−^ ionic charge and (**c**) TEA^+^ ionic charge stored on positive electrodes in the voltage range of 0~2.5 V. (**d**) Schematic illustrations of possible charge storage process in graphene nano-channels.

Figure 4a shows the electronic charge stored on the positive electrodes as a function of the increasing charging voltage. It was calculated by integrating the current intensity of current-time curves (Supplementary Information, Fig. S2) without the contribution of self-discharge. Figure 4b shows the BF_4_^−^ ionic charge stored on positive electrodes at different charging voltages, where the BF_4_^−^ ionic charge was calculated based on the relationship between anion population and NMR spectra intensity shown in Fig. 2a. As the charging voltage increased from 0 to 2.5 V, the BF_4_^−^ ionic charge (*i.e*., the population of BF_4_^−^ anions) within graphene nano-channels decreased firstly and then increased sharply with the charging voltages higher than 1.0 V.

In order to understand the migration of TEA^+^ cations in electrodes, the TEA^+^ ionic charge stored on positive electrodes was calculated based on the principle of charge conservation. Figure 4c shows the TEA^+^ ionic charge (*i.e*., the population of TEA^+^ cations) within the graphene channel decreased greatly at the charging voltage lower than 1.0 V. As discussed in the previous section, the expulsion of cations was the primary mechanisms of charge storage in graphene electrodes. Excess cations have been expelled from the graphene nano-channels upon charging to balance the electronic charge that accumulated in the graphene surface. Meanwhile, the population of anions decreased abnormally, possibly because amounts of BF_4_^−^ anions were taken out of the channel by the cations during the charging process. Similar observation was also reported by Luo *et al*.^36^, where a nonlinear behavior of both Na^+^ and BF_4_^−^ on negative charging above 0.6 V was observed. This abnormal phenomenon was caused by the competing effect between the ion-ion correlations and the ion-surface electrostatic interactions. In this case, the decreasing TEA^+^ cations concentration favors the dragging of BF_4_^−^ anions out of the graphene channels because of the strongly ion-ion correlations between electrolyte ions. As the charging voltage increased from 1.0 to 2.5 V, a gradually increase in the TEA^+^ ionic charge was observed, which means that a number of TEA^+^ cations were brought into graphene films by anions upon charging.

Figure 4d schematically shows the possible charge storage process in graphene nano-channels. At low charging voltages ranging from 0 V to 1.0 V, the charge storage process was driven by the expulsion of TEA^+^ cations. Meanwhile, a number of anions were taken out of graphene films by cations because of the ion-ion correlations between BF_4_^−^ anions and TEA^+^ cations (as schematically shown in the “stage 1”). An opposite phenomenon showed up at the charging voltages of 1.0 V~2.5 V. In such a voltage range, the charge storage process is dominated by the adsorption of BF_4_^−^ anions. At the same time, a number of TEA^+^ cations were taken into the graphene nano-channels by BF_4_^−^ anions upon charging because of the strongly ion-ion correlation electrolyte ions (as schematically shown in the “stage 2”). The charge storage in negative electrodes has the similar process with the increasing charging voltage (Supplementary Information, Fig. S3).

Two distinct charging stages were observed on the graphene-film based EDLCs charged at voltage values varying from 0 to 2.5 V. At low charging voltages (0~1.0 V), the expulsion of co-ions (TEA^+^ cations for positive electrodes, BF_4_^−^ anions for negative electrodes) played an important role in the charge storage process. In this stage, the co-ions were expelled from graphene nano-channels with the increasing of charging voltages. At a higher charging voltage (1.0~2.5 V), the charge storage process was dominated by the adsorption of counter-ions (BF_4_^−^ anions for positive electrodes, TEA^+^ cations for negative electrodes). Large numbers of counter-ions were adsorbed in to the graphene channels to balance the electronic charge that accumulated in the graphene surface. During the charging process, the abnormal decrease or increase in the number of counter-ions or co-ions within graphene channels arised from the strongly ion-ion correlation between cations and anions.

## Conclusions

In summary, ^11^B MAS-NMR measurements were performed on graphene-film based EDLCs. In neutral graphene films, the anions were preferentially adsorbed on graphene surfaces at relatively low electrolyte loading volumes (<50 μL). The ion population of weakly and strongly adsorbed anions increased as a function of electrolyte loadings, and partial desolvation of ion solvation shells was found at higher loading volumes (>100 μL). Unlike previous NMR studies on other porous carbons and CNTs, the number of strongly adsorbed anions was much more than that of the weakly adsorbed anions at each certain electrolyte loading, mainly due to the unique two-dimensional layered structure of graphene films. The NMR experiments carried out at different voltages showed that the charging process can be divided into two distinct charge storage stages for different voltage ranges (*i.e.*, ejection of co-ions at relatively low voltages and adsorption of counter-ions at higher voltages). The abnormal decrease or increase in the number of counter- or co-ions (*e.g.*, the abnormal decrease in the number of anions in the positive electrodes between 0 and 1.0 V) could be attributed to the ion-ion correlation between cations and anions. The results obtained in the current NMR measurements provide useful insights for advancing the optimization of graphene-film based EDLCs.

## Methods

### Preparation of Graphene Film

Graphite oxide (GO) powder (250 mg) synthesized by modified Hummer’s method was dispersed in deionized water (1 L) and ultrasonicated (FB15150, 300 W, Fisher Scientific) for 1.5 h. The resulting GO dispersion was then refluxed in a 95 °C oil bath for 2 h with 4 mL ammonia solution (ca. 25~28 wt% in water, Sinopharm Chemical Reagent Co. Ltd) and 206 μL hydrazine hydrate (85 wt% in water, Sinopharm Chemical Reagent Co. Ltd). Graphene film was fabricated by vacuum filtration of the resulting dispersion through a membrane filter of 0.22 μm in pore size, and dried in desiccator for a week before transferred to glovebox.

### Preparation of NMR samples

In an argon glovebox, a series amounts (50, 75, 100, 125, 150, 200 μL) of 1 M tetraethylammonium tetrafluoroborate (TEABF_4_, 99.0%, Sigma-aldrich Co.) in acetonitrile (ACN, 99.8%, Sigma-aldrich Co.) electrolyte were added into as-prepared graphene films (60 mg) and soaked for 12 h in an airtight container. Then these samples were taken out of the container and dried in argon environment for 10 min.

Graphene-film based EDLCs were assembled in a two electrode system with 1 M TEABF_4_/ACN electrolyte. In order to eliminate the effect of irreversible changes that could take place at the first few charging cycles, the EDLCs were cycled by galvanostatic charge-discharge cycle for 30 mins with charge/discharge rate of 1 A g^−1^. After that, the EDLCs were held at the desired constant voltages (*i.e*., 0, 0.5, 1.0, 1.5, 2.0, 2.5 V) for 30 min and then disassembled quickly within 1 min. Then the graphene films electrode materials were separated from the current collector and dried under argon environment for 10 mins. The electrochemical measurements were performed on an electrochemical workstation (PGSTAT302N, Metrohm Autolab B.V) at room temperature.

### NMR experiments

Slid-state NMR experiments were performed on a Bruker AVANCE III 400 MHz spectrometer operating at 128.42 MHz for ^11^B, and equipped with a double-resonance magic-angle spinning (MAS) probe, supporting MAS rotors of 3.2 mm outer diameter. ^11^B Slid-state NMR spectra were recorded at MAS frequency of 10 kHz, employing the direct excitation method with rf-nutaion frequency of 140 kHz for ^11^B. ^11^B chemical shift was referenced to NaH_4_B at −42.06 ppm. A recycle delay of 5 s was used to collect a total of 64 scans for each sample. NMR spectra with a spectral width of 608.37 ppm (78125.00 Hz) were recorded in all cases. To ensure the experimental parameters were proper in the current work, the spin-lattice (T_1_) relaxation time for ^11^B were measured with an inversion-recovery pulse sequence in separate experiments (see Fig. S4 in Supplementary Information for more details). All NMR spectra were fitted with mixed Gaussian/Lorentzian function lineshapes using the DMFIT software^53^. In order to estimate the errors caused by deconvolution, fits were repeated up to four times for each series of data^34^. The standard errors were calculated by the following equation:


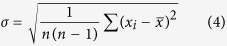


where *n* (=4) refers to the number of data points for each case, *x*_*i*_ is the value of these data point, and 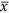 represents the average values of *x*_*i*_.

## Additional Information

**How to cite this article**: Li, K. *et al*. Solid-state NMR Study of Ion Adsorption and Charge Storage in Graphene Film Supercapacitor Electrodes. *Sci. Rep.* **6**, 39689; doi: 10.1038/srep39689 (2016).

**Publisher's note:** Springer Nature remains neutral with regard to jurisdictional claims in published maps and institutional affiliations.

## Supplementary Material

Supplementary Information
