# Supplementary material for: Solid-state NMR Study of Ion Adsorption and Charge Storage in Graphene Film Supercapacitor Electrodes
Source: Sci Rep. 2016 Dec 21;6:39689. doi: 10.1038/srep39689 (PMC5175154; doi:10.1038/srep39689)
Supplement: Supplementary Information [file srep39689-s1.doc]

Supplementary Information

Solid-state NMR Study of Ion Adsorption and Charge Storage in Graphene Film Supercapacitor Electrodes

Kecheng Li, Zheng Bo*, Jianhua Yan, and Kefa Cen

State Key Laboratory of Clean Energy Utilization, Institute for Thermal Power Engineering, College of Energy Engineering, Zhejiang University, Hangzhou, Zhejiang Province, 310027, China

Correspondence should be addressed to Zheng Bo (Email: bozh@zju.edu.cn; Tel: 86 571 87953290)

**1. NMR spectrum of crystalline TEABF_4_.**

**
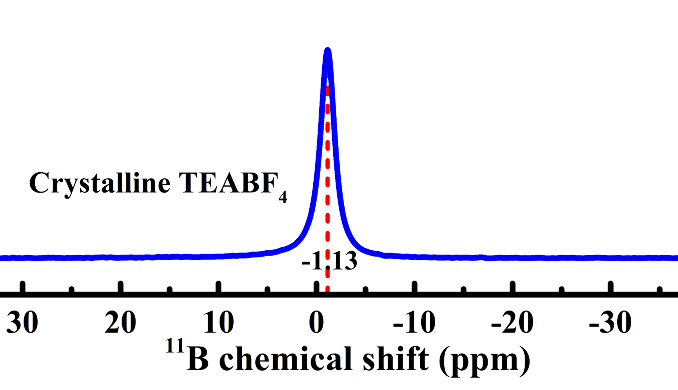
**

**Figure S1.** ^11^B MAS NMR spectrum of crystalline TEABF_4_.

**2. The voltage-time and current-time curves of graphene-film based EDLCs charged at different voltages.**

**
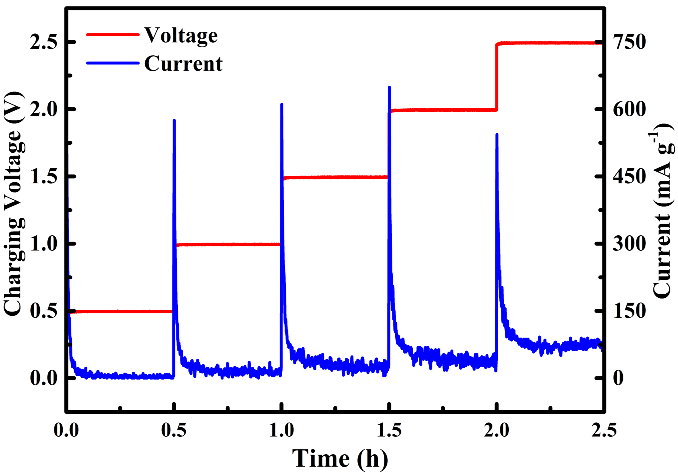
**

**Figure S2.** The voltage-time and current-time curves of graphene-film based EDLCs charged at different voltages. The EDLCs were tested by charging from 0 V to 2.5 V with a step of 0.5 V every 30 mins. The electronic charge at every voltage has been calculated by integrating the current intensity without the contribution of self-discharge.

**3. Ionic charge stored on negative electrodes in the voltage range of 0 ~ 2.5 V.**

**
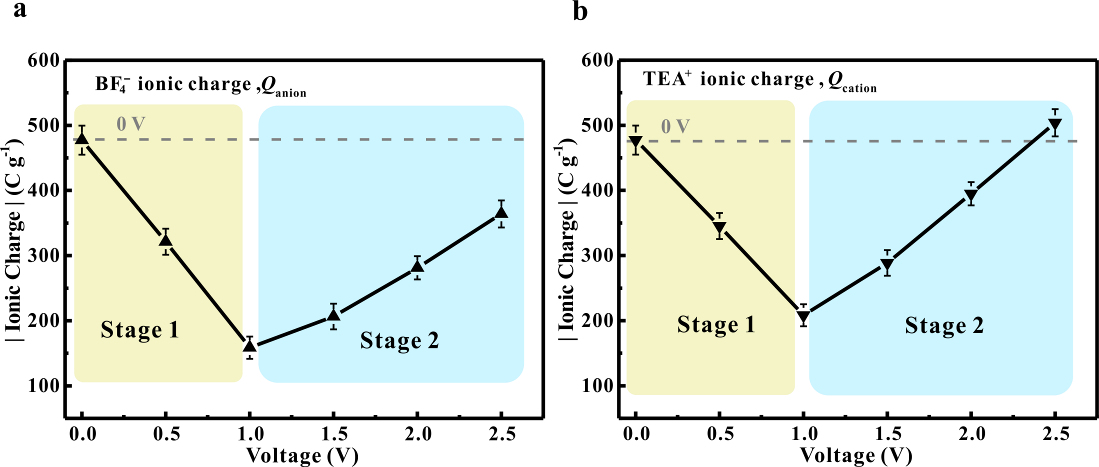
**

**Figure S3.** The a) BF_4_^-^ ionic charge and b) TEA^+^ ionic charge stored on negative electrodes in the voltage range of 0 ~ 2.5 V.

**4. ^11^B spin-lattice (T_1_) relaxation time for BF_4_^-^ anions in the current work.**

Fig. S4 (a) shows the ^11^B T_1_ time for BF_4_^-^ anions in neutral graphene films, a longer T_1_ value (0.307 s) was obtained at the electrolyte loading volume of 200 μL. Previous studies found that the T_1_ time for molecules adsorbed carbon surfaces are significantly reduced due to the interaction of the ^11^B nuclear spins with the unpaired spins at the Fermi level of the carbon conduction band^1,2^. For the experiment with 50 μL of electrolyte, the shorter T_1_ time for BF_4_^-^ anions may attribute to the strongly anion-carbon interactions between anions with graphene surface at lower electrolyte loadings. The ^11^B T_1_ relaxation time for BF_4_^-^ anions in positive (or negative) electrodes are further reduced compared with that of BF_4_^-^ anions in neutral graphene films (as shown in Fig. S4 b and c). This phenomenal can be attributed to the changes in the density of states at the Fermi level of carbons duo to ejection (or injection) of electrons in the electrodes^1,3^.

**
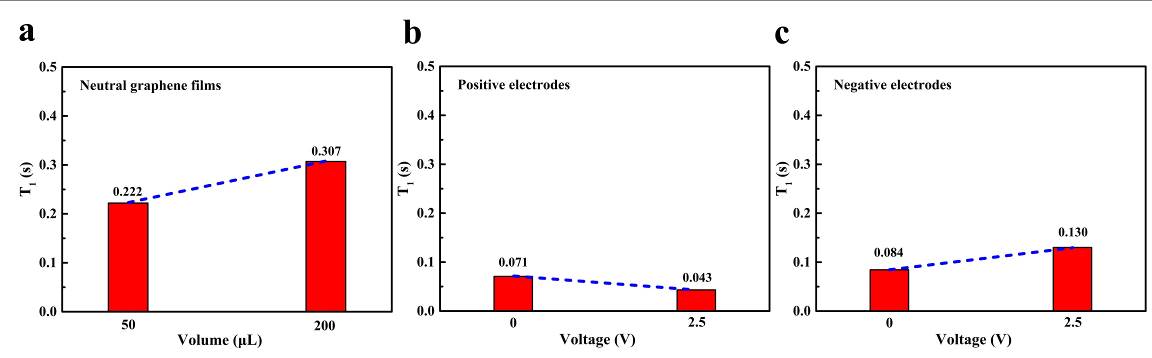
**

**Figure S4.** ^11^B spin-lattice (T_1_) times for BF_4_^-^ anions in a) neutral graphene films, b) positive electrodes and c) negative electrodes.

**Reference:**

1 Wang, H. *et al.* Real-time NMR studies of electrochemical double-layer capacitors. *J. Am. Chem. Soc.* **133**, 19270-19273 (2011).

2 Cosgrove, T., Copping, B. W. & Jarvis, R. A. Self-diffusion and nuclear spin relaxation of benzene adsorbed on graphitized carbon. *J. Colloid Interface Sci* **96**, 214-221 (1983).

3 Luo, Z. X. *et al.* Dehydration of Ions in Voltage-Gated Carbon Nanopores Observed by in Situ NMR. *J. Phys. Chem. Lett.* **6**, 5022-5026 (2015).
